# Possible functional links among brain- and skull-related genes selected in modern humans

**DOI:** 10.3389/fpsyg.2015.00794

**Published:** 2015-06-16

**Authors:** Antonio Benítez-Burraco, Cedric Boeckx

**Affiliations:** ^1^Department of Spanish Philology and its Didactics, University of Huelva, Huelva, Spain; ^2^Catalan Institute for Research and Advanced Studies, Barcelona, Spain; ^3^Department of Linguistics, Universitat de Barcelona, Barcelona, Spain

**Keywords:** language-ready brain, skull morphology, human evolution, Neanderthals/Denisovans, anatomically modern humans, AUTS2, FOXP2, RUNX2

## Abstract

The sequencing of the genomes from extinct hominins has revealed that changes in some brain-related genes have been selected after the split between anatomically-modern humans and Neanderthals/Denisovans. To date, no coherent view of these changes has been provided. Following a line of research we initiated in Boeckx and Benítez-Burraco (2014a), we hypothesize functional links among most of these genes and their products, based on the existing literature for each of the gene discussed. The genes we focus on are found mutated in different cognitive disorders affecting modern populations and their products are involved in skull and brain morphology, and neural connectivity. If our hypothesis turns out to be on the right track, it means that the changes affecting most of these proteins resulted in a more globular brain and ultimately brought about modern cognition, with its characteristic generativity and capacity to form and exploit cross-modular concepts, properties most clearly manifested in language.

## Introduction

The successful retrieval of ancient DNA and subsequent reconstruction of archaic human genomes (Pääbo, 2014a) surely qualify as a major breakthrough in our attempt to understand the evolution of our species. Ancient genomes constitute fossils of a new kind, potentially far more revealing than the more traditional fossils scientists have manipulated for decades. This research generates candidate genes to shed light on changes that led to the emergence of modern human cognitive capacities (Green et al., 2010; Meyer et al., 2012; Pääbo, 2014b; Prüfer et al., 2014). To date, no coherent view of these changes has been yet provided. In particular, no functional link between these changes has been established. In this paper, we would like to rely on the existing literature for each of the gene involved and hypothesize functional links among them. In other words, we claim that some of the key genetic changes related to the brain in modern humans did not occur independently of one another, but rather in a related fashion.

Before delving into these genes, we would like to briefly remind the reader that as important as it is to be able to provide a list of genes that affect the brain, we must ultimately be able to characterize what roles the cellular consequences of these gene products have for brain circuits and their dynamics and how those in turn give rise to specific cognitive phenotypes.

The psychological sciences already offer good characterizations of the human cognitive phenotype. Interestingly, for many of these, the scholars that put them forward tend to implicate our language capacity to a significant extent. For example, Spelke (2003) takes language to be crucial in the establishment of productive, systematic exchange of information across “core knowledge” systems shared with other species. Likewise, Marcus (2001) sees in language the clearest illustration of what makes our mind “algebraic” (as opposed to merely “symbolic”). There is indeed a growing consensus among scientists of various disciplines—from paleoanthropology and archeology (Mithen, 1996; Coolidge and Wynn, 2005) to philosophy (Carruthers, 2006; Pietroski, 2012) and linguistics (Reinhart, 2006; Boeckx, 2011)—that our brain’s language-readiness, understood not only as a communication system but also and perhaps most importantly as the generator of a particular conceptual format, is the central piece of the puzzle of our cognitive modernity.

If on the right track, the centrality of language in delimiting our cognitive phenotype would allow us to take advantage of the decades of results in formal linguistics to come up with a detailed, computationally explicit description of the “human cognome” (Poeppel, 2012)—the set of elementary mental representations and operations needed to generate the anlage of our rich cognitive life (e.g., Samuels, 2011; Boeckx, 2014). Taken as a whole, these elementary representations and operations would constitute the proper target of neurobiological investigations.

To those who have already ventured in this direction, it is clear that a successful marriage of the relevant disciplines will require a serious rethinking of the neurobiological foundations of language processing (Fedorenko and Thompson-Schill, 2014; Hagoort, 2014; Poeppel, 2014). The age of the “classical model” of brain and language, developed in the nineteenth century by pioneers like Broca, Wernicke, and Lichtheim is over. As reviewed in Petersson et al. (2012), the language network is more extended than the classical language regions and includes, next to Broca’s region, adjacent cortex in the left inferior and middle frontal region, as well as substantial parts of superior and middle temporal cortex, inferior parietal cortex, as well as subcortical structures such as the basal ganglia, the cerebellum, the hippocampus, and the thalamus.

As it slowly replaces the classical model, this new “neurobiology of language” (Poeppel et al., 2012) approximates distributed networks implicated in cognition more generally (working memory models, the default network, the multiple demand system, the global neuronal workspace model, etc.; see Boeckx and Benítez-Burraco, 2014a), thereby becoming a better candidate for the hypothesis that language-readiness is central to modern cognition, as opposed to just another encapsulated module of the mind.

It is with this background in mind that we want to examine the genetic changes that separate us from our extinct closest relatives, the Neanderthals and the Denisovans. To do so, we have decided to rely on our (Boeckx and Benítez-Burraco, 2014a) proposal, centered around *RUNX2*.

Briefly summarizing our (Boeckx and Benítez-Burraco, 2014a) paper, we put forward a set of genes involved in skull morphogenesis, thalamic development and the specification, migration and interconnection of GABAergic neurons within the forebrain. The most salient members of this set of genes were RUNX2, the *DLX* suite (in particular *DLX1*, *DLX2*, *DLX5*, *DLX6*), genes of the *BMP* family (*BMP2*, *BMP7*), and interacting partners like *SHH* and *FGF8*. We hypothesized that the evolutionary modification of this network may account for our species-specific globular head shape (compared to Neanderthals; Bruner, 2004; Gunz et al., 2010, 2012; Neubauer et al., 2010) and for the concomitant rewiring of different connections between cortical and sub-cortical (specifically, thalamic) structures, which we claimed provide the scaffolding for our species-specific mode of cognition (see Boeckx and Benítez-Burraco, 2014a, for details; see also Benítez-Burraco and Boeckx, 2015).

Once this initial set of genes was established, we decided to examine potential links between these genes and the rich body of work on *FOXP2* (a robust candidate for language disorders in our species) and its network (Vernes et al., 2011; Graham and Fisher, 2013). The results were presented in Boeckx and Benítez-Burraco (2014b). There, we paid special attention to *ROBO1*, which appears to be critical to aspects of language such as vocal learning (Pfenning et al., 2014). As we will highlight below, the new set of genes discussed below further strengthen the links between our initial set of genes and the *FOXP2* network.

**FIGURE 1:**
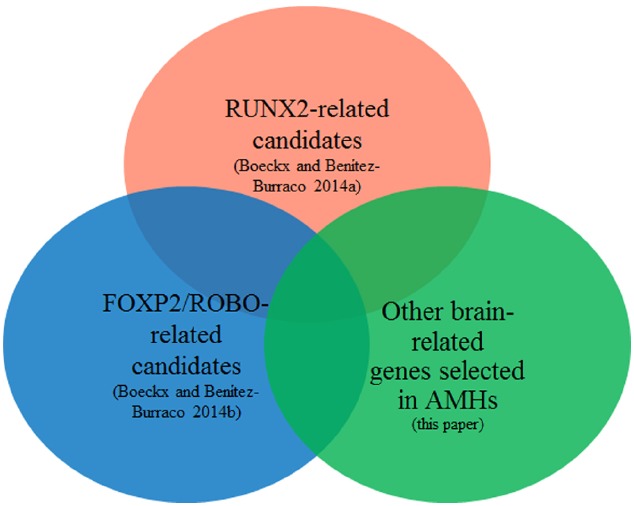
**Objective of the present study: focus on the intersection of the sets of genes highlighted in Boeckx and Benítez-Burraco (2014a,b) with the currently available list of genes showing signs of positive selection in anatomically modern humans**.

Figure 1 provides a schematic representation of our strategy.

At this point we would like to highlight a few considerations that will bear in the discussion to follow. Specifically, the three considerations we now turn to helped us select both the genes discussed in the next section, and the information we present for each.

First, we think that a detailed investigation of the genetic underpinnings of our species-specific brain-growth trajectory that gives rise to a more globular braincase configuration ought to pay attention as well to changes in the visual system, and their cognitive implications, something which we did not do in our original (Boeckx and Benítez-Burraco, 2014a) study. The globularization of the brain is likely to affect the occipital lobe, which provides the roots of the visual system. In addition, a recent study by Pearce et al. (2013), focusing on the fact that Neanderthals had larger eyes than our species, suggests that more of their brain was devoted to seeing in the long, dark nights in Europe, at the expense of high-level cognitive processing. This is so because larger eyes entail a much larger visual processing area at the back of their brains. In other words, more of the Neanderthal brain would have been dedicated to vision and body control. A reduction of the visual area in anatomically modern humans (AMHs) has been independently supported by Sherwood et al. (2008), and it may have led to an expansion of the parietal region, and a re-allocation of the computational power of the pulvinar, the part of the dorsal thalamus that modulates cortical visual processing (Saalmann et al., 2012), in service of other cognitive domains, such as language. The fact that visual deficits either predispose toward or protect against mental diseases like schizophrenia for which language is crucially implicated (Silverstein et al., 2013; Leivada and Boeckx, 2014), suggest that the visual system and visual cognition ought to play a role in any account of the brain’s language-readiness and the emergence of modern cognition more generally. Some of the findings reported below are meant to bear directly on this hypothesis.

Second, an important aspect of language-readiness—the ability to form and maintain long-distance connections across the whole brain to form and exploit cross-modular concepts—is likely to require the recruitment of thalamic nuclei such as the medio-dorsal nucleus and the pulvinar in addition to neocortical areas. Accordingly, both cortical and subcortical reconfigurations must be taken into account. In particular, we think that it is crucial to focus on new sources of inhibition in light of neo-cortical and pulvinar expansion. In this context, Rakic’s hypothesis concerning new sources of interneurons due to molecular changes (Letinic and Rakic, 2001; Rakic, 2009; Geschwind and Rakic, 2013) strike us as particularly relevant. Incidentally, our concern for sources of inhibition as well as the establishment and maintenance of long-distance cortical connections naturally led us to pay special attention to mental diseases like autism, where the factors just mentioned appear to be targeted (Just et al., 2004; Yizhar et al., 2011; Khan et al., 2013; Le Magueresse and Monyer, 2013; Zikopoulos and Barbas, 2013). Hence the frequent mention of autism below (see also Benítez-Burraco and Boeckx, 2015).

Third, in addition to the precise establishment of cortico-thalamic and thalamocortical connections acting as cognitive pacemakers, it is also important to examine any changes concerning interhemispheric connectivity (Theofanopoulou, 2015). Although neurolinguistic studies have traditionally been lopsided (focus on the left-hemisphere), recent reviews reporting progress in the neurobiology of language emphasize the importance of the right hemisphere and interhemispheric integration for language (see Petersson et al., 2012; Poeppel et al., 2012; Poeppel, 2014; Theofanopoulou, 2015). This also led us to pay attention to mental conditions in which interhemispheric connectivity patterns and language abilities are altered: again autism turns out to be a key source of information (Verly et al., 2014).

Armed with all these considerations, we turn to brain-related changes revealed by discoveries based on archaic human genomes, and examine their possible implications for the brain organization (and head shape) of AMHs.

## Methods

Our goal in this paper is to establish potential functional connections with the initial gene set discussed in Boeckx and Benítez-Burraco (2014a), the FOXP2 interactome as currently known (reviewed in Boeckx and Benítez-Burraco, 2014b), and the literature on selected genes in AMHs.

Our *modus operandi* has been as follows:

a. We searched the literature via PubMed^1^ looking for symptoms of interest for us linked to the mutation of the “brain-related” genes frequently mentioned in publications addressing the list of changes selected for in AMHs (e.g., Crisci et al., 2011; Lalueza-Fox, 2013; Lombard et al., 2013). We also relied on the most recent and more exhaustive list of genes showing signals of positive selection in AMHs given in Pääbo (2014b, Table S1). Some of the key search terms used were: “language disorder,” “cognitive disorder,” “intellectual disability,” “syntax deficit,” “semantic deficit,” “phonological deficit,” “speech deficit,” “dyslexia,” “schizophrenia,” “autism,” “autism spectrum disorder (ASD),” etc.
b. When a gene of interest was identified, we looked for potential links with the set of genes as advanced in Boeckx and Benítez-Burraco (2014a) via String 9.1.^2^ String 9.1 predicts direct/physical and indirect/functional associations between proteins that derive from four sources: genomic context, high-throughput experiments, conserved coexpression, and the knowledge previously gained from text mining (Franceschini et al., 2013). We considered links with a confidence value above 0.0400. This score means that one has a 40% probability that a predicted link exists between two enzymes in the same metabolic map in the KEGG database^3^. We used this medium confidence value because the highest one (0.700) was too restrictive and we did not get many links (as is typical of any approaches relying on String). Importantly, we checked all the predicted links against the existing literature and discarded all the links for which we could not find strong evidence, in order to achieve a reliable set of candidates for our potential network.
c. We did not systematically gather information beyond more than three connection levels. Thus, our limit was of the sort “A is connected to B which is in turn connected to C.” We considered additional levels only if the identified genes were more closely connected to some other gene(s) of interest previously reviewed. For example, “A is connected to B which is in turn connected to C which is in turn connected to D (but D is connected to *RUNX2*).”
d. We also used PubMed to look for genetic information related to brain areas, circuits, neural processes, neurotransmitters, etc., of interest for us; specifically, the neurological considerations of Boeckx and Benítez-Burraco (2014a) highlighted in Section “Introduction,” as well as those neural substrates emphasized in the skull and brain literature. For this, we used search terms like “thalamus,” “thalamo-cortical connection,” “interhemispheric connection,” etc.

Because the amount of literature on single genes varies greatly, we did not necessarily discard any gene because the number of manuscripts was under a value we may have pre-selected.

In addition to PubMed, we also relied on the following databases, which we also used for our (Boeckx and Benítez-Burraco, 2014a) paper.

(1) The microarray database of the Allen Brain Atlas.^4^
(2) The Prenatal LMD Microarray search engine.^5^
(3) The Developmental Transcriptome browser^6^ of the Allen Brain Atlas.
(4) OMIM for the linguistic and cognitive deficits linked to the mutation of genes of interest.^7^

Additionally, as we did in Boeckx and Benítez-Burraco (2014b), we have exploited the information provided in Kuhlwilm et al. (2013), where 691 genes were found to be differentially expressed after *RUNX2* transfection in neuroblastomic SH-SY5Y cells, as well as the detailed FOXP2 targets list provided in Konopka et al. (2009), Spiteri et al. (2007), and Vernes et al. (2007).

While collecting information on each new gene of interest, we paid special attention to its potential role not only in language, but also vision, in the establishment of cortico-thalamic and thalamo-cortical connections, and in the balance between excitation and inhibition in the context of brain growth. This focus is reflected in the information we present in the next section.

## *RUNX2* and Brain-Related Genes Selected in AMHs

Because of the centrality of *RUNX2* in our (Boeckx and Benítez-Burraco, 2014a) proposal, we decided to start this section by briefly reviewing the nature and relevance of this gene, focusing on information that was not available at the time we completed our initial study.

### RUNX2

There is solid evidence of a selective sweep in *RUNX2* after our split from Neanderthals (Green et al., 2010). Although the aminoacid sequence of RUNX2 is conserved between AMHs, Neanderthals and Denisovans, its promoter region presents two derived alleles in modern humans (Perdomo-Sabogal et al., 2014). As Perdomo-Sabogal et al. (2014) point out, this might have caused gene regulatory changes with implications for cranial, skeletal and bone development in modern humans, given the well-established fact that *RUNX2* controls different aspects of the morphology of the upper body and the cranium: closure of cranial sutures, clavicle development, rib cage formation, and dental growth (Stein et al., 2004). In particular, the action of RUNX2 under the control of DLX5 (Holleville et al., 2007) and TLE1 (Wang et al., 2004) appears crucial for the integration of the parietal bone (Depew et al., 1999; Stephens, 2006), heavily implicated in the globularization of the AMH brain/skull (Bruner, 2004).

Although *RUNX2* does not figure among the canonical “brain-related” genes in publications addressing the list of changes selected for in AMHs (e.g., Crisci et al., 2011; Lalueza-Fox, 2013; Lombard et al., 2013), unlike genes like *AUTS2* or *DYRK1A*, which we discuss below, we think that it should be included among genes of great relevance for our cognitive phenotype. Here is why.

To begin with, Jeong et al. (2008) and Reale et al. (2013) demonstrate that *RUNX2* expression plays an important role at the brain level (thalamus, hypothalamus, hippocampus), and the gene is repeatedly mentioned as a candidate for several mental diseases, including schizophrenia, and bipolar disorders (Benes et al., 2007; Subburaju and Benes, 2012; Talkowski et al., 2012; Ruzicka et al., 2015). Recently, *RUNX2* has been shown to interact with several key candidate genes for autism, specifically with *SMURF1* (De Rubeis et al., 2014), a gene involved in the regulation of axonogenesis (Cheng et al., 2011; Kannan et al., 2012).

More indirectly, *RUNX2* (and “skull-related” genes more generally) provides crucial information about brain growth and connectivity, as the very signals sent to build the brain case, and thus influencing *RUNX2* expression, come from the same signaling molecules that have been independently argued to be responsible for brain organization (WNT, FGF, SHH, DLX, etc.). Especially in the first year of life, when globularity is established in AMHs, brain and skull exhibit an extremely tight fit (Lieberman, 2011), which may well explain why mental retardation and craniofacial dysmorphism co-occur, even more so that one suspects (see Boeckx and Benítez-Burraco, 2014a, for review). Studies on other species such as dogs demonstrate how skull shape and brain connectivity influence one another (Roberts et al., 2010), so we feel entitled to apply this logic to the case of AMHs. Incidentally, this also applies to *FOXP2*, which interacts with genes that not only affect brain connectivity but also craniofacial bones (e.g., *ROR2*, *COL9A1*, Konopka et al., 2009). As a matter of fact, direct physical interaction between *RUNX2* and *FOXP2* has recently been experimentally demonstrated (Zhao et al., 2015).

There is also mounting evidence in favor of bone affecting cognition. As reviewed in Oury et al. (2013), the regulation of bone mass exerted by subcortical structures like the hypothalamus (Driessler and Baldock, 2010), suggests the existence of bone-derived signals modulating this regulation or other functions of the brain. As Oury et al. (2013) show, the osteoblast-derived hormone osteocalcin crosses the blood–brain barrier, binds to neurons of the brainstem, midbrain, and hippocampus, enhances the synthesis of monoamine neurotransmitters, inhibits GABA synthesis, prevents anxiety and depression, and favors learning and memory independently of its other metabolic functions at the bone level. In addition to these postnatal functions, maternal osteocalcin crosses the placenta during pregnancy and prevents neuronal apoptosis before embryos synthesize this hormone. Thus, the authors conclude, the skeleton via osteocalcin influences cognition and contributes to the maternal influence on fetal brain development.

Interestingly, *RUNX2* is deeply implicated in the regulation of osteocalcin, including at the brain level (Vladimirova et al., 2008). RUNX2 has been shown to interact with the 1alpha,25-dihydroxyvitamin D3 receptor (encoded by *VDR*) to up-regulate rat *Bglap* (encoding osteocalcin) expression in osteoblastic cells (Paredes et al., 2004). We take this interaction between RUNX2 and VDR to be extremely relevant. Stephens and Morrison (2014) have shown that RUNX2 and VDR, which becomes active when bound by its ligand 1,25-dihydroxyvitamin D3 (VD3), unite to cooperatively regulate the expression of numerous genes. Among the genes identified are *SPAG5*, which is among the genes selected by AMHs (Green et al., 2010), and to which we return below, and also *SRGAP3*. The latter has been shown to play an important role in severe mental retardation and absence of speech (Endris et al., 2002). Srgap3^–/–^ mice present a neurodevelopmental disorder with schizophrenia-related intermediate phenotypes (Waltereit et al., 2012), and the gene has been shown to be implicated in childhood onset schizophrenia in humans (Wilson et al., 2011). In addition, Srgap2 acts through Srgap3 to modulate neuronal differentiation and neurite outgrowth of mouse neuroblastoma cells (Ma et al., 2013). We highlight this fact because *SRGAP2* is a gene that has duplicated three times in humans (Sudmant et al., 2010). One of the duplicated forms encodes a truncated form of the protein that binds to the non-truncated protein encoded by the ancestral gene (Dennis et al., 2012). When expressed in mouse neuronal precursor cells, the truncated protein variant results in increased density of longer neuronal spines (Charrier et al., 2012). More generally, *SRGAP3*-mediated cytoskeletal reorganization has an important influence on a variety of neurodevelopmental processes, which may be required for normal cognitive function. (Endris et al., 2002; Bacon et al., 2013; Radaei et al., 2014). In particular, SRGAP2 and SRGAP3 interact with ROBO1 and affect the SLIT/ROBO pathway (Wong et al., 2001), which is critical for a range of brain connectivity patterns, including in the context of language (see Boeckx and Benítez-Burraco, 2014b, for review, and especially Pfenning et al., 2014, where SLIT-mediated interaction between FOXP2/foxp2 and ROBO1/robo1 is claimed to be critical to the establishment of vocal learning pathways across species).

Like *RUNX2*, *SRGAP3* is heavily expressed in the hippocampus (Waltereit et al., 2012). Hippocampal network alterations are well attested in a variety of human cognitive diseases, such as schizophrenia (Heckers, 2001), bipolar disorder (Frey et al., 2007), Alzheimer’s disease (Schuff et al., 2009), and Down syndrome (Pennington et al., 2003). In the context of Alzheimer’s, it may be worth pointing out that RUNX2 interacts with APOE (Vaes et al., 2006; Kuhlwilm et al., 2013), a gene that has been consistently related to some of the metabolic changes that allowed bigger brains, and eventually enhanced cognitive capacities, to evolve within hominins (Bufill and Carbonell, 2006), even if one of its alleles (*ε*4) appears to predispose its bearers to Alzheimer’s. Incidentally, the link between *RUNX2* and *APOE* may well reinforce the hypothesis put forth by Bruner and Jacobs (2013) that Alzheimer’s disease is intimately connected to the globularization of the AMH skull/brain.

In a similar vein, Shen and Christakos (2005) have shown that the Vdr, Runx2, and the Notch signaling pathway cooperate in the transcriptional regulation of *Spp1*, which encodes osteopontin, which is also important for the brain. Osteopontin-deficient mice have been shown to suffer from thalamic neurodegeneration (Schroeter et al., 2006). A disregulation of osteopontin is known to cause intracranial arteriovenous malformations (Xu et al., 2012).

Finally, vitamin D (Patrick and Ames, 2014) and other RUNX2 interacting partners (e.g., CREB; Oury et al., 2010) also regulate serotonin synthesis. Not only does brain serotonin regulate bone mass through its release in ventromedial hypothalamic neurons, it also affects thalamocortical axonal system development (van Kleef et al., 2012), and has been claimed to be implicated in autism (Patrick and Ames, 2014).

As a final note, we wish to point out that brain expression of Foxp2 was shown to be significantly altered by vitamin D deficiency (Hawes et al., 2015).

Having discussed *RUNX2*, we now turn our attention to more canonical “brain-related” genes that show evidence of selection in AMHs. We begin with *AUTS2*.

### AUTS2

According to Green et al. (2010) the first half of *AUTS2* (roughly, the section of the gene encompassing exons 1–4 [chr7:68,662,946-69,274,862 (hg18)]) displays the strongest signal of positive selection in AMHs compared to Neanderthals (within this region, two of the 293 SNPs that are ancestral in Neanderthals are coding variants, although they are not fixed in AMHs). Perhaps for this reason *AUTS2* has been subject of recent attention (see Oksenberg and Ahituv, 2013, for review).

Three non-coding intronic regions of interest in evolutionary terms have been found within *AUTS2*: the human accelerated region HAR31, located in intron 4 (Prabhakar et al., 2006), and the human accelerated conserved non-coding sequences (haCNSs) HACNS 369 and HACNS 174, located in introns 1 and 4, respectively (Pollard et al., 2006). Interestingly, these regions contain enhancers that seem to be active in the brain. Within the region under selective sweep Oksenberg et al. (2013) found six enhancers that show expression in the brain and four mouse enhancers that are active in the midbrain and the eye. Some of these enhancers are also found inside the HAR or HACNS regions. It may be the case that some of the enhancers encompassing the *AUTS2* regulatory landscape affect other genes, perhaps to some of the genes located within the Williams Syndrome critical region (see Oksenberg and Ahituv, 2013, for discussion). Williams syndrome is a developmental disorder involving cognitive and linguistic deficits (see Mervis and Becerra, 2007; Martens et al., 2008, for reviews), which results from a microdeletion in one copy of the chromosome 7 that affects roughly two dozen genes (Korenberg et al., 2008). Among these genes one finds *GTF2I*, related to craniofacial abnormalities and cognitive problems (Morris et al., 2003; Tassabehji et al., 2005), which represses *RUNX2* (Lazebnik et al., 2009) and is a functional partner of *USF1* (Roy et al., 1997), both of them central pieces in our (Boeckx and Benítez-Burraco, 2014a) paper. Incidentally, the regulatory region of *USF1* has undergone 30 fixed or high frequency changes after our split from Denisovans (Meyer et al., 2012).

Of particular interest to us is the fact that *AUTS2* is a candidate for several neurodevelopmental disorders, including ASD, intellectual disability, and developmental delay (see Oksenberg and Ahituv, 2013, for discussion). Cognitive impairment can co-occur with epilepsy, skeletal abnormalities, brain malformations, and/or dysmorphic features—the so-called *AUTS2 syndrome* (Beunders et al., 2013). Copy-number variation and genome wide associations studies have related *AUTS2* with other ASD-independent disordered conditions, like schizoaffective disorder (Hamshere et al., 2009), bipolar disorder (Hattori et al., 2009), differential processing speed (Luciano et al., 2011), or even dyslexia (Girirajan et al., 2011). According to Talkowski et al. (2012) the AUTS2 *locus* is also linked to microcephaly, macrocephaly, ataxia, visual impairment, motor delay, or Rubinstein–Taybi syndrome. Recently, Amarillo et al. (2014) have found a deletion encompassing exon 6 of *AUTS2* in a subject with severe speech and language problems (the individual was unable to make full sentences at the age of 4;6). During growth *AUTS2* is expressed in the human brain throughout the telencephalon (primarily in the frontal, parietal, and temporal lobes), but also in some other regions, including the cerebellum and the basal ganglia (caudate and putamen nuclei; Sultana et al., 2002; Lepagnol-Bestel et al., 2008). The gene is also highly expressed in the ocular tissues (Wagner et al., 2013). In mice, *Auts2* has proved to be expressed in the developing thalamus, the cerebellum and the cerebral cortex, in neurons of several types, including glutamatergic, GABAergic, and dopaminergic neurons (Bedogni et al., 2010a). Interestingly, *Auts2* is expressed in the thalamus both prenatally (in the dorsal thalamus) and postnatally (in the anterior thalamic nuclei and in ventrolateral/ventromedial nuclei). In the cortex *Auts2* is only expressed in the frontal areas after birth (Bedogni et al., 2010a). The knockdown of *auts2* in zebrafish causes a decrease in neuronal cells which results in microcephaly. Craniofacial abnormalities are also observed, as well as motor problems (plausibly due to a reduced number of motor neurons and/or sensory neurons) and a reduced size of the eye (Beunders et al., 2013; Oksenberg et al., 2013).

In sum, available evidence points to *AUTS2* as an important gene for neuro-cranial development and human evolution. However, the putative link between this gene, the changes that occurred at the brain level to give rise to AMHs, and the emergence of modern cognition and the language abilities we have, remain unclear. What follows is an attempt to offer a hypothesis that could guide future experimental investigations seeking to address this issue.

Our attempt starts with the noteworthy finding that *AUTS2* figures among the genes found to be differentially expressed after *RUNX2* transfection in neuroblastomic SH-SY5Y cells (Kuhlwilm et al., 2013). Even more so is the fact that both *Runx2* and *Foxp2* are found among *Auts2* regulatory targets (Oksenberg et al., 2014), as is *Cntnap2*. *CNTNAP2* is a well-known FOXP2 target (Vernes et al., 2008) and a candidate for language delay and language impairment (Petrin et al., 2010; Sehested et al., 2010), intellectual disability (Gregor et al., 2011), and autism (Alarcón et al., 2008; Bakkaloglu et al., 2008), The AMH CNTNAP2 exhibits a fixed change (Ile345Val) compared to the Denisovan protein (Meyer et al., 2012). Moreover, CNTNAP2 is related to NFASC, a protein involved in neurite outgrowth and the formation of postsynaptic components (Kriebel et al., 2012) that shows a fixed change (T987A) in AMHs compared to Neanderthals/Denisovans (Pääbo, 2014b, Table S1).

Interestingly, AUTS2 regulation of *RUNX2* also affects *CBL*. Mutations in the latter gene cause Noonan syndrome-like disorder, a condition characterized by facial dysmorphism, a reduced growth, and a variety of cognitive deficits, among other symptoms (Martinelli et al., 2010). Of interest to us is the fact that *CBL* is located in a region showing signal of a strong selective sweep (20-fold enrichment over random) in AMHs compared to Altai Neanderthals (Prüfer et al., 2014).

Needless to say, AUTS2 has been associated with many other proteins that play a key role at the brain level. Many of these are also candidate for autism or for developmental disorders affecting cognitive/language abilities. These include TBR1, RELN, SATB2, GTF2I, ZMAT3, or PRC1 (reviewed by Oksenberg and Ahituv, 2013). We will discuss some of these genes in detail here, but we will focus in their connections with both RUNX2 and FOXP2, and explore additional connections with other AMH-selected genes.

### TBR1

In mice *Auts2* and *Tbr1* are coexpressed mostly in glutamatergic neurons of the forebrain (Bedogni et al., 2010a). In fact, *Auts2* is a direct target of Tbr1 in the developing neocortex (Bedogni et al., 2010b). Microdeletions causing the disruption of *TBR1* give rise to severe speech and language deficits, autistic-like problems, and moderate to severe intellectual disability, while larger deletions encompassing *TBR1* cause delayed or absent speech and language and intellectual disability (Palumbo et al., 2012, 2014; Traylor et al., 2012). Microcephaly is also a reported (Palumbo et al., 2012), plausibly due to the functional link between *TBR1* and *ASPM*, a robust candidate for microcephaly, and candidate for positive selection in human lineage (Bond et al., 2002; Zhang, 2003).

*TBR1* has been shown to interact with *FOXP2* (Enard et al., 2009; Deriziotis et al., 2014), as Ferland et al. (2003) had anticipated in mice. Specifically, Ferland et al. (2003) had speculated that disruption of *Tbr1* disrupts *Foxp2* expression in layer VI of the cortex, thereby altering the projections of layer VI neurons to the dorsal thalamus. *TBR1* indeed plays a key role in the organization of the corticothalamic tracts, which originate mostly from layer VI of the cortex (Hevner et al., 2001). Srinivasan et al. (2012) have shown that in mice the expression of *Tbr1* consistently correlates with subcortical axons that innervate the dorsal thalamus. After birth, *Trb1* expression is upregulated in different upper-layer neurons and this upregulation is required for the expression of *Auts2* (Bedogni et al., 2010b; Srinivasan et al., 2012). The knockout of the gene almost abolishes neocortical connectivity with the thalamus [which is, among other things, a critical aspect of the language-ready brain, if we are right in our (Boeckx and Benítez-Burraco, 2014a) hypothesis], and misroutes corticothalamic axons of neurons from layer VI and the subplate toward the spinal cord (Han et al., 2011; McKenna et al., 2011).

In addition to this role in corticofugal connectivity, *Tbr1* is also involved in the establishment of intercortical connections. Specifically, callosal axons fail to cross the midline in the absence of Tbr1 (Hevner et al., 2001). Interestingly, the integrity of the corpus callosum is frequently reported to be affected in people suffering from ASD (Kumar et al., 2010; Shukla et al., 2010; Nagae et al., 2012); also, as we advanced in Section “Introduction,” a decrease in interhemispheric connections seems to be a hallmark of this neurodevelopmental condition (Shukla et al., 2010; Ingalhalikar et al., 2011; Weinstein et al., 2011). Moreover, in mice *Tbr1* haploinsufficiency also results in defective axonal projections of amygdalar neurons, which give rise to a deficit in ultrasonic vocalization, social interaction, and associative memory and cognitive flexibility (Huang et al., 2014).

### FEZF2

Much of the function performed by *TBR1* seemingly results from its effect on *FEZF2* expression. *Fezf2* has been claimed to be a key orchestrator of gene activity giving rise to neuronal subtypes (Lodato et al., 2014). In mice the knockdown of *Tbr1* results in the ectopic upregulation of *Fezf2* (Han et al., 2011; McKenna et al., 2011). In fishes, *fezf2* is one of the components (together with *six3*, *shh*, *irx1b*, and *wnt1*) of the gene suite controlling the relative size of the telencephalon versus the thalamus and ultimately, the differences in brain architecture among species (Sylvester et al., 2010). In mice *Fezf2* is expressed exclusively in the corticofugal projection neurons of the deep cortical layers, mostly in subcerebral neurons from layer V, and also, but less strongly, in corticothalamic neurons from layer VI (Chen et al., 2005; Molyneaux et al., 2005). *Fezf2* directs the differentiation of neural stem cells in the subventricular zone toward specific cortical phenotypes (Zuccotti et al., 2014), and it has been shown to regulate the gene suite that define the glutamatergic corticospinal neurons (Lodato et al., 2014) and the fate of pyramidal neurons in deep layers of the cortex.

In the absence of *Fezf2* expression neurons express *Satb2* and project to other regions of the cortex. Moreover, the number of interneurons of several subtypes—critical for the correct balance between excitation and inhibition in the neocortex—becomes reduced in layer V of the cortex (Lodato et al., 2011). Conversely, the presence of Fezf2 causes neurons to project to subcortical regions (Chen et al., 2008). Specifically, the gene controls the development and the corticothalamic projections of neurons from layer VI (Chen et al., 2005; Molyneaux et al., 2005). Interestingly, *FEZF2* (and also its partner *CTIP2*) is highly expressed in von Economo neurons (Cobos and Seeley, 2013). These neurons, located in layer V of the anterior cingulate and the fronto-insular cortices, are important for social behavior and are only found in species that are able of self-awareness (great apes, elephants, or cetaceans; Butti et al., 2009; Allman et al., 2010). Cortical areas containing von Economo neurons form a network of frontoparietal functional connectivity, encompassing four different sub-networks, involved in saliency detection, sensory-motor behavior, and attention (Cauda et al., 2013). Significantly, autistics show a higher ratio of Von Economo neurons to pyramidal neurons in the frontoinsular cortex, which has been related to an enhanced interoception (Santos et al., 2011).

Not surprisingly, then, *FEZF2*, like *TBR1*, is a candidate for autism (Wang et al., 2009). Interestingly, the SNP found by Wang et al. (2013), to be associated with this condition, located within the proximal promoter region, represents a reversion to the ancestral non-primate allele (the primate allele has been strongly selected in our clade). Interestingly also, FEZF2 is required for the expression of *FOXP2* (Molyneaux et al., 2005).

### DYRK1A

Another promising partner of *TBR1* is *DYRK1A*, a gene often mentioned alongside *AUTS2* in the context of human evolution, given that it contains a region identified to have strong signals of selective sweep in AMHs compared to Neanderthals (Green et al., 2010). TRB1 regulates *RELN* (Chen et al., 2002), an important gene controlling neural migration and also a candidate for lissencephaly with language loss (Hong et al., 2000) and for autism (Wang et al., 2014). In turn, *RELN* is upregulated by FOXO1 (Daly et al., 2004). *FOXO1* is a target of both RUNX2 (Kuhlwilm et al., 2013) and FOXP2 (Vernes et al., 2011), and encodes a protein that is phosphorylated by DYRK1A (Huang and Tindall, 2007).

*DYRK1A* is located within the Down Syndrome Critical Region on chromosome 21. Mutations on *DYRK1A* give rise to microcephaly, facial dysmorphisms, mental retardation, and absence of speech (van Bon et al., 2011; Courcet et al., 2012). The gene is expressed during development, but also in the adult brain, and it seems to be involved in learning and memory (Hämmerle et al., 2003). This role is explained by the effect of DYRK1A on synaptic plasticity and on the expression of genes encoding GABAergic and glutaminergic related proteins. This effect ultimately alters the balance between excitation and inhibition, which we believe is very important for cognitive function. Specifically, *DYRK1A* overexpression, as observed in Down syndrome, affects pathways involved in synaptogenesis and synaptic plasticity, and moves the excitation/inhibition balance toward inhibition (Souchet et al., 2014).

Interestingly, increasing expression of *Dyrk1a* in mice upregulates *GAD1* (Souchet et al., 2014). *GAD1* is a target of FOXP2 (Konopka et al., 2009). Moreover, RUNX2, DLX1, and DLX2, three key genes in our (Boeckx and Benítez-Burraco, 2014a) hypothesis concerning globularity and the emergence of the language-ready brain, are also key components of the GAD1 regulatory network, which is important for the normal development of GABAergic neurons within the hippocampus (Pleasure et al., 2000; Ruzicka et al., 2015). Additionally, in mice *Dyrk1a* has been shown to play a central role in the balance between cortical and thalamic neurons: in particular, *Dyrk1a* copy number seems to be directly related to neuron density in the thalamic nuclei and indirectly related to neuron density in the cortex (Guedj et al., 2012).

Finally, DYRK1A directly phosphorylates SIRT1 and also promotes deacetylation of TP53. SIRT1 is involved in the regulation of different neural processes, including neural precursor activity and differentiation (Saharan et al., 2013) and axon formation and elongation (Li et al., 2013a). As we discussed in Boeckx and Benítez-Burraco (2014a), a potential link exists between *FOXP2* and *RUNX2* via SIRT1. Moreover, SIRT1 directly regulates RUNX2: it both upregulates *RUNX2* and deacetylates RUNX2, ultimately promoting osteoblast differentiation (Shakibaei et al., 2012; Srivastava et al., 2012). In this context, it may be worth mentioning that SIRT1 is an effector of several genes that are under selection in modern populations and that show non-fixed changes in their coding regions compared to Neanderthals and Denisovans, like *BAZ2A* and *NR1H2* (Prüfer et al., 2014). Interestingly also, SIRT1 regulates some of the genes controlling biological noise, like H2A.Z (Baptista et al., 2013). This may have helped buffer the molecular noise resulting from the changes occurred in the RUNX2 network and that brought about the language-ready brain (Benítez-Burraco, 2015).

Regarding TP53, it is a candidate for schizophrenia (Ni et al., 2005). A non-fixed change (Pro72–Arg72) has been found in the AMH protein compared to Neanderthals/Denisovans (Paskulin et al., 2012). Last, but not least, as we also review in Boeckx and Benítez-Burraco (2014a), *TP53* is related to many of the genes encompassing the language-ready brain network, including *SIRT1*, *USF1* (mentioned above), *CDH1*, *ASPM* (also mentioned above), and *PTEN*.

### SATB2

An additional partner of *TBR1* and *AUTS2* we wish to highlight in this part of the paper is *SATB2*. Together with *Fezf2*, *Ctip2*, and *Tbr1*, *Satb2* controls the identity of stereotypic projections in the cortex. Specifically, in conjunction with Ctip2, Satb2 regulates *Tbr1* expression in neurons from cortical layers II to V in order to produce callosal projections. Together with Fezf2, Satb2 regulates *Auts2* (Srinivasan et al., 2012). Sequence and copy number variations in *SATB2* have been found in patients with ASD, intellectual disability, and developmental and language delays, as well as craniofacial defects (see Kwan, 2013 and especially Liedén et al., 2014, for discussion). Accordingly, *SATB2* is also involved in osteogenesis, where it directly interacts with *RUNX2* (Hassan et al., 2010). For example, lentiviral-mediated-Satb2-transduced cells from the mouse bone marrow stroma overproduce Satb2 and upregulates *Runx2* (Gong et al., 2014). Finally, an interesting link also exists between *SATB2* and *FOXP2*. Hence, Dobreva et al. (2006) have found that Satb2 represses the expression of several Hox genes, including *Hoxa2*. This gene encodes an inhibitor of bone formation and regulator of branchial arch patterning. According to Konopka et al. (2009) *HOX2A* is among FOXP2’s targets. Also, the phenotype linked to chromosome 2q32-q33 deletions and to the haploinsufficiency of *SATB2* can be mimicked by the haploinsufficiency of *GTF3C3*, a gene that is also a target of FOXP2 (Konopka et al., 2009).

In the context of Satb2, we wish to point out that a strong Gli3 binding region is located just over 100 kb ≤ of *Satb2* in neural tissue (Vokes et al., 2007). Gli3 interacts with Shh during thalamic development (Haddad-Tóvolli et al., 2012). Moreover, Gli3 regulates calvarial suture development by controlling Bmp-Smad signaling, which integrates a Dlx5/Runx2-II cascade (Tanimoto et al., 2012). Actually, mutations in *GLI3* have been found in people affected by Greig cephalopolysyndactyly syndrome, a condition in which craniosynostosis is an important feature (Debeer et al., 2003). Interestingly, most (∼98%) of Altaic Neanderthals and Denisovans had a different sequence in *GLI3* compared to AMHs: while the latter retained the ancestral sequence, the former gained a non-synonymous change that appears to be mildly disruptive (Castellano et al., 2014). Given the role of Satb2 in the establishment of callosal projections, it is also interesting that Gli3 controls corpus callosum formation by positioning midline guideposts during telencephalic patterning (Magnani et al., 2014), altering expressions of *Slit1/2*, *Fgf8*, and Wnt/β-catenin, genes we discussed at length in Boeckx and Benítez-Burraco (2014a).

### ZBTB20

At this point we would like to briefly mention another gene, *ZBTB20*, which also shows signs of selection in AMHs (Green et al., 2010). We find this gene of interest because it defines a hippocampal neuronal identity through direct repression of genes that control projection neuron development in the isocortex, among which we found *Fezf2*, *Satb2*, *Tbr1*, and *Foxp2* (Nielsen et al., 2014). In addition, although we do not know of any direct evidence for an interaction between ZBTB20 and RUNX2 in the hippocampus, we note that Kuhlwilm et al. (2013) found several genes of the ZBTB family to be differentially expressed after *RUNX2* transfection in neuroblastomic SH-SY5Y cells.

### PAX6

Although we are not aware of any signal of positive selection of *PAX6* in AMHs, there are robust links between them and *RUNX2*, *FOXP2*, *AUTS2*, and other genes discussed above.

*PAX6* encodes a transcription factor involved in the development of the eye and the brain. Although perhaps best known as the master regulator or master selector of eye development (Gehring and Ikeo, 1999), in the brain it affects the process of glutamatergic neuron differentiation (Kim et al., 2014). It has been suggested that changes in *PAX6* expression may underlie the imbalance in excitatory/inhibitory neuronal activity in the autistic brain because of its involvement in glutamatergic differentiation during development (Kim et al., 2014), and indeed mutations on this gene have been linked to some forms of ASD (Maekawa et al., 2009). Importantly, *PAX6* is coexpressed with *AUTS2* in the ventricular and subventricular regions (Bedogni et al., 2010a).

*PAX6* has been shown to be involved in interhemispheric transfer (Bamiou et al., 2004), to the extent that mutations in this gene result in working memory problems (Bamiou et al., 2007). Moreover, *PAX6* plays an important role in the development of thalamic connections. Specifically, mammillo-thalamic tracts were missing in *Pax6* mutant mice (Valverde et al., 2000). Repression of *Shh* by Pax6 regulates diencephalic patterning by controlling the central diencephalic organizer (Caballero et al., 2014). Additionally, *Pax6* regulates the orientation and mode of cell division of progenitors in the mouse cerebral cortex, influencing as it does cell adhesion during cortical development (Tyas et al., 2003). In this context, it interacts with many of the genes discussed in the previous subsection, and gives rise to cortical layering abnormalities when knocked-out in mice (e.g., Tuoc et al., 2009). Pax6 plays as well a crucial role at the level of the cortical hem, which regulates both the size and patterning of the cortex (Caronia-Brown et al., 2014), but also at the cortical anti-hem (Subramanian et al., 2009). Positioned as a mirror image of the cortical hem, along the lateral margin of the cortical primordium, the cortical anti-hem is identified by gene expression for three epidermal growth factor family members, Tgfβ, Nrg1, and Nrg3, as well as two other signaling molecules, Fgf7 and the secreted Wnt antagonist Sfrp2. The anti-hem is lost in mice homozygous for the *Pax6* mutation. It is worth mentioning in this context that both *FGF7* and *NRG3* are among the genes that show signals of a selective sweep in AMHs compared to Neanderthals (Green et al., 2010; Prüfer et al., 2014).

A heterozygous mutation of *PAX6* has been shown in individuals with aniridia and deficits in executive and social cognition. Structural abnormalities of gray matter were reported in the anterior cingulate cortex, the cerebellum and the medial temporal lobe, as well as white matter deficits in corpus callosum. Functional MRI demonstrated reduced activation of fronto-striato-thalamic systems during performance of overt verbal fluency and non-sense sentence completion; the most consistent abnormality of verbal executive activation was located in the thalamus (Ellison-Wright et al., 2004). Eye problems associated with *PAX6* deficiencies are also worth bearing in mind in light of the potential cognitive relevance of eye size in Neanderthals and AMHs (Pearce et al., 2013), as we reviewed in the first section of the paper. Anophthalmia and microphthalmia are repeatedly reported in this context (Verma and FitzPatrick, 2007), and are often associated with mental diseases like schizophrenia where language is affected (Leivada and Boeckx, 2014).

In zebrafish the knockdown of *pax6* disrupts the expression of both *arx* and *foxp2* (Coutinho et al., 2011). *ARX* is actually a target of *FOXP2* (Konopka et al., 2009; Vernes et al., 2011). *ARX* directly controls *SLIT2* in the proper formation of the thalamus. *ARX* is in turn directly regulated by DLX genes in the developing forebrain, and contributes to the tangential migration of GABAergic interneurons (Colasante et al., 2008). Analysis of transcriptional codes for zebrafish dopaminergic neurons has revealed essential functions of *arx* in prethalamic dopaminergic neuron development (Filippi et al., 2012). In human mutations of *ARX* give rise to mental retardation and interneuronopathies (Quillé et al., 2011), including lissencephaly (Kitamura et al., 2002) and agenesis of the corpus callosum (Proud et al., 1992) which are features of interest in the context of this paper.

Like *AUTS2*, *PAX6* figures among the genes found to be differentially expressed after *RUNX2* transfection, although in a different cell line (HepG2; Kuhlwilm et al., 2013). Moreover, among the PAX6 target genes and enhancers listed by Coutinho et al. (2011) we have found several genes of great interest to us, including *FOXP2*, *ASCL1*, *DLX1*, *DLX2*, *DLX5*, *DLX6, GBX2*, and *GLI3*, all of which we discussed in Boeckx and Benítez-Burraco (2014a) and above. In addition, *PAX6* interacts with *TBR1* at the level of neurogenesis (Englund et al., 2005).

Interestingly, among PAX6 target genes, we have also found *POU3F2*, this meaning that both *FOXP2* and its effector *POU3F2* are regulated by PAX6. *POU3F2* has been associated with haCNSs (Miller et al., 2014). There exists an AMH-specific substitution in intron 8 of *FOXP2* that affects a binding site for POU3F2 (Maricic et al., 2013). The derived allele has proved to be less efficient in activating transcription (Maricic et al., 2013). It is possible then that *FOXP2* expression in Neanderthals and Denisovans was higher than in AMHs as a consequence of this change. In this context it is worth pointing out that overexpression of *FOXP2* in humans has been related to autism via *MET*, a putative risk factor for this condition (Mukamel et al., 2011). *POU3F2* has been linked to bipolar disorder (Mühleisen et al., 2014), but also to developmental and language delay, intellectual disability, schizophrenia and ASD (Lin et al., 2011). *POU3F2* encodes a transcription factor that controls dopamine and serotonin synthesis (Nasu et al., 2014) and also the regulation of the upper-layer neuronal migration and identity during the development of the neocortex (McEvilly et al., 2002; Sugitani et al., 2002; Domínguez et al., 2013). POU3F2 is also expressed at the level of the corpus callosum (Katoh and Katoh, 2009), and it interacts with PQBP1, in turn linked to developmental delay and microcephaly (Li et al., 2013b) and to intellectual disability (Wang et al., 2013).

### SPAG5

As pointed out above, the interaction between RUNX2 and VDR promotes the expression of *SPAG5*, which is among the genes selected in AMHs (Green et al., 2010) The gene encodes a protein needed for the correct function of mitotic spindles (it is required for centrosome integrity and the maintenance of sister chromatid cohesion during mitosis; Thein et al., 2007), but also for the regulation of apoptosis induced by cell stress (Thedieck et al., 2013). At the brain level SPAG5 helps PAX6 to regulate the sequential symmetric and asymmetric cell division of neuronal precursors. Hence, in mice the downregulation of *Spag5* mimics the knockout of *Pax6*, which greatly alter the orientation and mode of cell division in the cerebral cortex and which results in an increased number of progenitors with morphological defects and an excess of daughter cells with asymmetric fates (Asami et al., 2011). Interestingly, *SPAG5* seems to be upregulated in people suffering from Down syndrome with cryptorchidism (Salemi et al., 2013). Moreover, in the retina SPAG5 interacts with the isoform B of USH2A, the main candidate for Usher syndrome, a condition involving combined deaf-blindness (Kersten et al., 2012). There exists an intriguing link between Usher syndrome and cognitive disorders involving language deficits, including psychosis, and schizophrenia (Domanico et al., 2012; see Leivada and Boeckx, 2014, for a more detailed discussion), which we think is worth studying further if our current reflections are on the right track.

**FIGURE 2:**
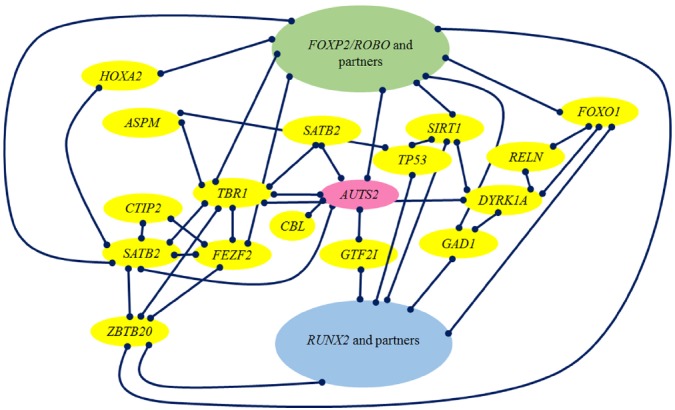
**Schematic representation of the strongest links discussed in this section**.

Figure 2 provides a graphic representation of the links we have wished to highlight in this section between *RUNX2*, brain-related genes selected in AMHs like *AUTS2* and *DYRK1A*, their closest partners *TBR1*, *FEZF2*, and *SATB2*, *FOXP2* and *PAX6*.

## Other Potential Genes of Interest

In this section, we wish to highlight additional changes in genes relevant for brain function that occurred after the split between AMHs and Neanderthals/Denisovans and that could be related to some of the genes considered so far, as well as those in Boeckx and Benítez-Burraco (2014a,b). These genes may help to make more robust the links between our initial gene set and the two gene sets we have reviewed above. This is why we discuss them in the remainder of this section.

To begin with, a region upstream *MEF2A* shows signals of recent positive selection in AMHs (Somel et al., 2013). Boeckx and Benítez-Burraco (2014a)) piece we hypothesized that the concomitant change in *MEF2A* expression could have contributed to a delayed peak expression and an increased level expression of synaptic genes in the prefrontal cortex of modern humans, and eventually to a less fast cortical synaptic development in AMHs compared to Neanderthals. We also found that *MEF2A* is functionally related to some of the components of our network, including *USF1*, *BMP2*, and *SIRT1*.

Also, *ERBB4* (which is linked to our language-ready-brain core set of candidates via *BMP2*) is a receptor of *NRG3*, which, as already mentioned, is among the genes that show signals of a strong selective sweep in AMHs compared to Neanderthals (Green et al., 2010; Prüfer et al., 2014). In the chicken cerebellum Erbb4 works jointly with Pten (Sakakibara and Horwitz, 2006), a gene that is coexpressed with *Katna1*, which in turn encodes a protein involved in the reorganization of cellular microtubule arrays (Selçuk et al., 2013). KATNA1 bears a fixed change (A343T) in AMHs compared to Neanderthals/Denisovans (Pääbo, 2014b, Table S1).

Moreover, FLNA interacts with ITGB4 (Travis et al., 2004), a protein that shows two fixed changes (T1689A and H1748R) in AMHs compared to Neanderthals/Denisovans (Pääbo, 2014b, Table S1). FLNA binds CMIP (Grimbert et al., 2004), a candidate for specific language impairment (Newbury et al., 2009), which contributes to regulate the assembly of synaptic complexes and/or neural migration (Grimbert et al., 2003). FLNA also interacts with a protein called CDC42 (Leung et al., 2010), required for proper migration of cortical interneurons (Katayama et al., 2013). Two effectors of CDC42 are worth considering: CDC42EP4 and ARHGAP32. Approximately 1kb within the *CDC42EP4* is hypermethylated in AMHs compared to Denisovans (Gokhman et al., 2014). Moreover, ARHGAP32 bears a fixed change (E1489D) in AMHs compared to Denisovans (Meyer et al., 2012). This latter protein promotes axon growth downstream CDH1 by interacting with SMURF1, which, as already mentioned above, is in turn related to RUNX2 and a substrate of CDH1 (Kannan et al., 2012).

Additionally, a protein called NCAM1, related to working memory performance (Bisaz et al., 2013) and to neuropsychiatric conditions like schizophrenia, bipolar disorder and Alzheimer’s disease (Atz et al., 2007), interacts with VCAM1, a cell surface glycoprotein that shows a fixed change (D414G) in AMHs compared to Neanderthals/Denisovans (Pääbo, 2014b, Table S1). *NCAM1* is a potential target of RUNX2 (Kuhlwilm et al., 2013), but also of FOXP2 (Konopka et al., 2009). Also *DISC1* is a target of FOXP2 (Walker et al., 2012) and a robust candidate for schizophrenia (Miyoshi et al., 2004). It is also highly expressed in the embryonic corpus callosum at a critical time for callosal formation (Osbun et al., 2011; Theofanopoulou, 2015). DISC1 interacts with PCNT, a protein of the centrosome and a candidate for dyslexia (Poelmans et al., 2011). *PCNT* is mentioned by Green et al. (2010) among the 11 genes that show non-synonymous and non-fixed substitution changes in their coding sequences compared to Neanderthals.

In the context of changes in the visual system and the establishment of the modern language-ready brain, it is worth noting that the protein SOLH bears two aminoacidic changes in AMHs (Green et al., 2010). Little is known about SOLH, but the little we know (e.g., Kamei et al., 1998) strike us as relevant. *SOLH* is a human homolog of the *Drosophila* small optic lobes gene. As its name suggests, mutations in the *Drosophila* gene cause a severe reduction in the neuropiles of the medulla and lobula complexes of the adult optic lobes. Northern analysis of human tissues revealed a *SOLH* transcript of approximately 5 kb that was strongest in the human brain. Kamei et al. (1998) mapped the *SOLH* gene to chromosome 16p13.3 by in situ hybridization. SOLH is a candidate gene for CATM syndrome (hereditary cataracts with microphthalmia), which maps in this region.

Of direct interest to us is the fact that *SOLH* is a target of FOXP2. It is among the genes whose expression level changes in a Foxp2-dependent manner in the E16 mouse (Vernes et al., 2011), and is reported as a direct target of FOXP2 in the human inferior frontal cortex (Spiteri et al., 2007). Although the action of *FOXP2* in the brain is often tied to fine motor production and auditory perception (Kurt et al., 2012), several authors have claimed that it is not limited to these (e.g., Campbell et al., 2009). Links between *Foxp2* expression and the visual system have in fact been documented (Iwai et al., 2013) and they may be worth exploring further in the context of cross-modal plasticity (e.g., Horng et al., 2009).

Finally, some other genes of interest are *NCOA6*, which encodes an interactor of both EP300 and CREBBP, and which bears a M823I substitution in AMHs compared to Neanderthals/Denisovans (Pääbo, 2014b, Table S1); *ANAPC10*, one partner of *CDH1* (Nourry et al., 2004), which shows signals of a selective sweep in AMHs compared to Altai Neanderthals (Prüfer et al., 2014); and *CBL*, which is located in a region showing signal of a strong selective sweep (20-fold enrichment over random) in AMHs compared to Altai Neanderthals (Prüfer et al., 2014) EP300, CREBBP, CDH1, and CBL are also relevant for the language-ready brain (see Boeckx and Benítez-Burraco, 2014b for a detailed discussion).

**FIGURE 3:**
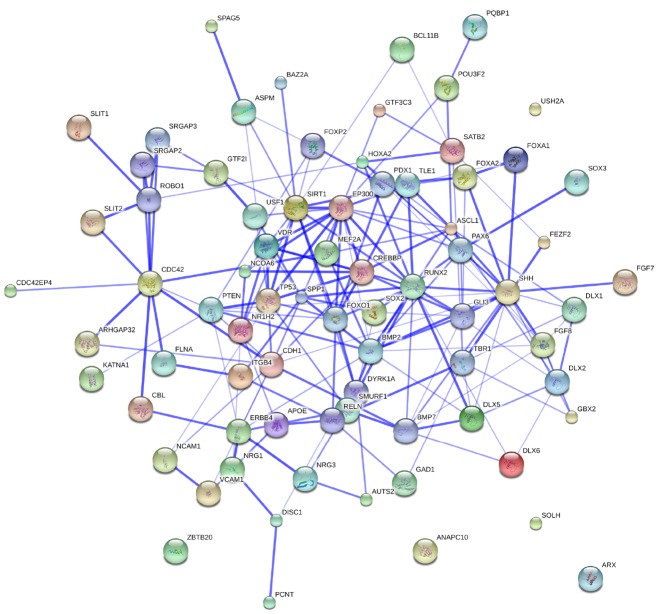
**The whole set of genes discussed in this paper.** The network was generated by String 9.1. The medium confidence value was 0.0400. Nodes representing the proteins encompassing the network are colored randomly. In this confidence view, stronger associations between proteins are represented by thicker lines. The figure does not represent a fully connected graph, but readers are asked to bear in mind that String 9.1 predicts associations between proteins that derive from a limited set of databases. The material discussed in the main text lead us to suspect connections that String does not generate (see Figure 2; although we wish to note that just adding a few genes, not discussed in this paper, yield a fully connected graph). It should be emphasized that the nature of String 9.1 is essentially predictive, and not explanatory. Although we have confirmed all the links we discuss here in the literature, they need to be confirmed at the brain level and in relation to language. Additionally, the diagram only represents the potential connectivity between the involved proteins, but this has to be mapped onto particular biochemical networks, signaling pathways, cellular properties, aspects of neuronal function, or cell-types of interest that can be confidently related to aspects of language development and function.

Figure 3 provides an overview of the genes discussed here, and some of the key interactions already derivable from the literature.

## Conclusion

The genomic revolution has led to a dramatic increase of information of great relevance to those interested in shedding light to what made us human. *SRGAP2* and *FOXP2* were the first identified members of a postulated set of genes that may have changed some of their functions (particularly, those related to brain growth and function) some moment between 2 or 3 million years ago and the present time as human ancestors grew larger brains and started to use these brains in new ways to perform complex tasks. But they are the first of a long list that remains to be explored in depth (recent other findings include the role of ARHGAP11B, Florio et al., 2015). We have focused on a few genes in this paper, and hope to have highlighted interesting connections that we hope future experiments will confirm. Our choice was guided by the hypothesis we put forth in our (Boeckx and Benítez-Burraco, 2014a) article, according to which the emergence of our species-specific language-ready brain ought to be understood in light of the developmental changes expressed at the levels of brain morphology and neural connectivity that gave us a more globular braincase configuration. As we argued in our earlier work, this globularization affected not only the shape of the skull, but also the cortico-subcortical connections (see Boeckx and Benítez-Burraco, 2014b piece). It also likely affected the visual system. If on the right track, the picture we have painted here suggests that there were coordinated changes tied to the emergence of modern cognition and language. Specifically, we think it is of great interest that the genes discussed here map onto specific types of neurons (GABAergic), particular brain structures (specific cortical layers, thalamic nuclei), specific physiological process (balance between inhibition and excitation), certain developmental processes (inter and interhemispheric axon pathfinding) and, when mutated, give rise to several disorders with recurrent endophenotypes. It is also of great interest that several of the genes discussed in the present paper have been examined for expression in the zebra finch brain and are reported in the public brain gene expression database (ZEBrA^8^), some of which representing markers of specific vocal nuclei or broader telencephalic zones generally associated with higher cognitive skills. Certainly, the number of functional links important for language evolution is becoming bigger as time goes by. Nonetheless, it has stopped being a mere list of binary interactions and has become instead a putative network that leads to testable predictions. We have proceeded here along standard lines: we have made our big network in a piecemeal fashion, by adding new pieces to our original, small core network. At the same time, the protein changes we highlight in our paper and the functional consequences we hypothesize for them should be viewed as instances of the sort of evolutionary kluge or complex reconfiguration (and ulterior adaptation) of ancestral systems that prompted the emergence of language as a cognitive faculty (Fisher and Marcus, 2006).

We wish to highlight from the onset the limitations of attempts like ours to perform literature-based assembly of protein–protein and gene-regulatory networks. We are aware that the robustness of each of the connections hypothesized here must be tested. Specifically, it is important to prove that the fixed coding changes in AMH proteins we review have impacted on their function. Importantly, although we have focused on the strongest links found in the literature, these links (often binary) have to be properly evaluated in order to know if they are actually biologically significant and meaningful regarding skull and brain development and function, and cognitive and linguistic abilities. We are aware that some types of evidence are stronger than other types. For example, data on direct protein:protein interactions is much more telling than, say, gene family membership. Even so, it should be proved that the involved proteins are expressed in the same brain region or cell type at an equivalent developmental stage (a point that is often overlooked by *in silico* tools). As we made explicit above we have tried to properly curate the data in order to rely on the strongest evidence only. We also acknowledge that the literature and the datasets we have relied on may be incomplete or biased because of the unavoidable focus on some genes as opposed to others. One should not forget that absence of evidence is not evidence of absence regarding protein–protein or DNA-protein interactions. It is also clear that the attested links for intensely studied proteins will always be more salient and more numerous than for less studied proteins. So, our main aim is to provide a list of potential candidates that can be employed as a useful starting point for future investigations. We think that for all its limitations, our type of research can offer valuable insights at this early stage of research in cognitive biology.

### Conflict of Interest Statement

The authors declare that the research was conducted in the absence of any commercial or financial relationships that could be construed as a potential conflict of interest.

## Footnotes

1 http://www.ncbi.nlm.nih.gov/pubmed

2 http://string-db.org/

3 http://www.genome.jp/kegg/pathway.html

4 http://human.brain-map.org/microarray/search

5 http://www.brainspan.org/lcm/search/index.html

6 http://www.brainspan.org/rnaseq/search/index.html

7 http://www.ncbi.nlm.nih.gov/omim/

8 www.zebrafinchatlas.org
